# Advancing a machine learning-based decision support tool for pre-hospital assessment of dyspnoea by emergency medical service clinicians: a retrospective observational study

**DOI:** 10.1186/s12873-024-01166-9

**Published:** 2025-01-05

**Authors:** Wivica Kauppi, Henrik Imberg, Johan Herlitz, Oskar Molin, Christer Axelsson, Carl Magnusson

**Affiliations:** 1 PreHospen- Centre for Prehospital Research, Faculty of Caring Science, Work Life and Social Welfare, University of Borås, Borås, SE- 501 90 Sweden; 2 Faculty of Caring Science, Work Life and Social Welfare, University of Borås, Borås, SE- 501 90 Sweden; 3 Statistiska Konsultgruppen Sweden, Gothenburg, Sweden; 4 Department of Molecular and Clinical Medicine, Institute of Medicine, Sahlgrenska Academy, University of Gothenburg, Gothenburg, Sweden; 5 Department of Prehospital Emergency Care, Sahlgrenska University Hospital, Gothenburg, Sweden

**Keywords:** Dyspnoea, Serious adverse event, Prehospital, Ambulance, Emergency medical services, Machine learning, Decision support tool

## Abstract

**Background:**

In Sweden with about 10 million inhabitants, there are about one million primary ambulance missions every year. Among them, around 10% are assessed by Emergency Medical Service (EMS) clinicians with the primary symptom of dyspnoea. The risk of death among these patients has been reported to be remarkably high, at 11,1% and 13,2%. The aim was to develop a Machine Learning (ML) model to provide support in assessing patients in pre-hospital settings and to compare them with established triage tools.

**Methods:**

This was a retrospective observational study including 6,354 patients who called the Swedish emergency telephone number (112) between January and December 2017. Patients presenting with the main symptom of dyspnoea were included which were recruited from two EMS organisations in Göteborg and Södra Älvsborg. Serious Adverse Event (SAE) was used as outcome, defined as any of the following:1) death within 30 days after call for an ambulance, 2) a final diagnosis defined as time-sensitive, 3) admitted to intensive care unit, or 4) readmission within 72 h and admitted to hospital receiving a final time-sensitive diagnosis. Logistic regression, LASSO logistic regression and gradient boosting were compared to the Rapid Emergency Triage and Treatment System for Adults (RETTS-A) and National Early Warning Score2 (NEWS2) with respect to discrimination and calibration of predictions. Eighty percent (80%) of the data was used for model development and 20% for model validation.

**Results:**

All ML models showed better performance than RETTS-A and NEWS2 with respect to all evaluated performance metrics. The gradient boosting algorithm had the overall best performance, with excellent calibration of the predictions, and consistently showed higher sensitivity to detect SAE than the other methods. The ROC AUC on test data increased from 0.73 (95% CI 0.70–0.76) with RETTS-A to 0.81 (95% CI 0.78–0.84) using gradient boosting.

**Conclusions:**

Among 6,354 ambulance missions caused by patients suffering from dyspnoea, an ML method using gradient boosting demonstrated excellent performance for predicting SAE, with substantial improvement over the more established methods RETTS-A and NEWS2.

**Supplementary Information:**

The online version contains supplementary material available at 10.1186/s12873-024-01166-9.

## Background

In Sweden, which has a population of roughly 10 million, there are nearly one million primary ambulance missions annually. Among these missions, approximately 10% involve patients where dyspnoea is identified as the primary symptom by Emergency Medical Service (EMS) clinicians, making it a frequent cause of ambulance calls in the country [1]. These patients have been reported to suffer from a variety of diseases some of which are benign whereas others are more harmful [2, 3]. Overall, the risk of death during the subsequent 30 days has been reported to be as high as 11,1% and 13,2% among patients who suffer from dyspnoea [2, 4]. In comparison, patients brought to the hospital by ambulance for severe trauma or chest pain have been noted to have a 30-day mortality rate of 3.6% and 2.1%, respectively [5, 6]. Although it is reasonable to assume that a relatively large proportion of patients with dyspnoea have an underlying time-sensitive condition [7] and therefore require a transport to hospital, some may not require hospitalization and may as well benefit from another level of care [8, 9]. A time-sensitive condition refers to a condition that is responsive to treatment, where the timing of intervention is critical to improving the outcome. In such cases, earlier initiation of treatment significantly enhances the prognosis [7]. Studies within the field of EMS specifically have explored various approaches to improve the prediction of SAEs and optimize patient care in pre-hospital settings. For instance, in the same patient cohort we described risk factors for underlying time-sensitive diagnoses and predictors of 30-day mortality [10]. Additionally, combining the Acute Dyspnoea Scale with vital parameters (VP) in the pre-hospital assessment of patients with dyspnoea has been shown to enhance SAE prediction [11]. This highlights the importance of dyspnoea severity, which is further described as a critical factor in predicting mortality outcomes for patients hospitalized with conditions such as acute heart failure [12]. There is a need for further knowledge regarding clinical variables that can predict a SAE among patients assessed by EMS clinicians in the pre-hospital setting with dyspnoea as the primary symptom. Findings using Machine Learning (ML) indicate that dyspnoea, identified across all levels of care, is a critical variable for predicting a SAE [13]. The utilization of Artificial Intelligence (AI) and ML is rapidly developing overall, and also in pre-hospital settings is still in its growing phase [14]. Recent studies highlight the potential of ML to support patient assessment in pre-hospital settings [15], enabling timely interventions to prevent critical events [16]. Given these advancements, in this study we developed ML models to predict the risk of a SAE among patients with dyspnoea assessed as the main symptom by EMS clinicians, and compared the performance of these models with current clinically available decisions tools: RETTS-A [17] and NEWS2 [18].

## Methods

### Study design

This was a retrospective observational study based on a consecutive review of EMS and hospital patient records. The study included all patients served by two EMS organisations who called the Swedish emergency telephone number (112) between January and December 2017. Upon receiving the call, an ambulance was dispatched to the scene, where patients presenting with dyspnoea as the main symptom were assessed by EMS clinicians. Further details can be found in Kauppi et al. (2020) [2].

### Setting

**Fig. 1:**
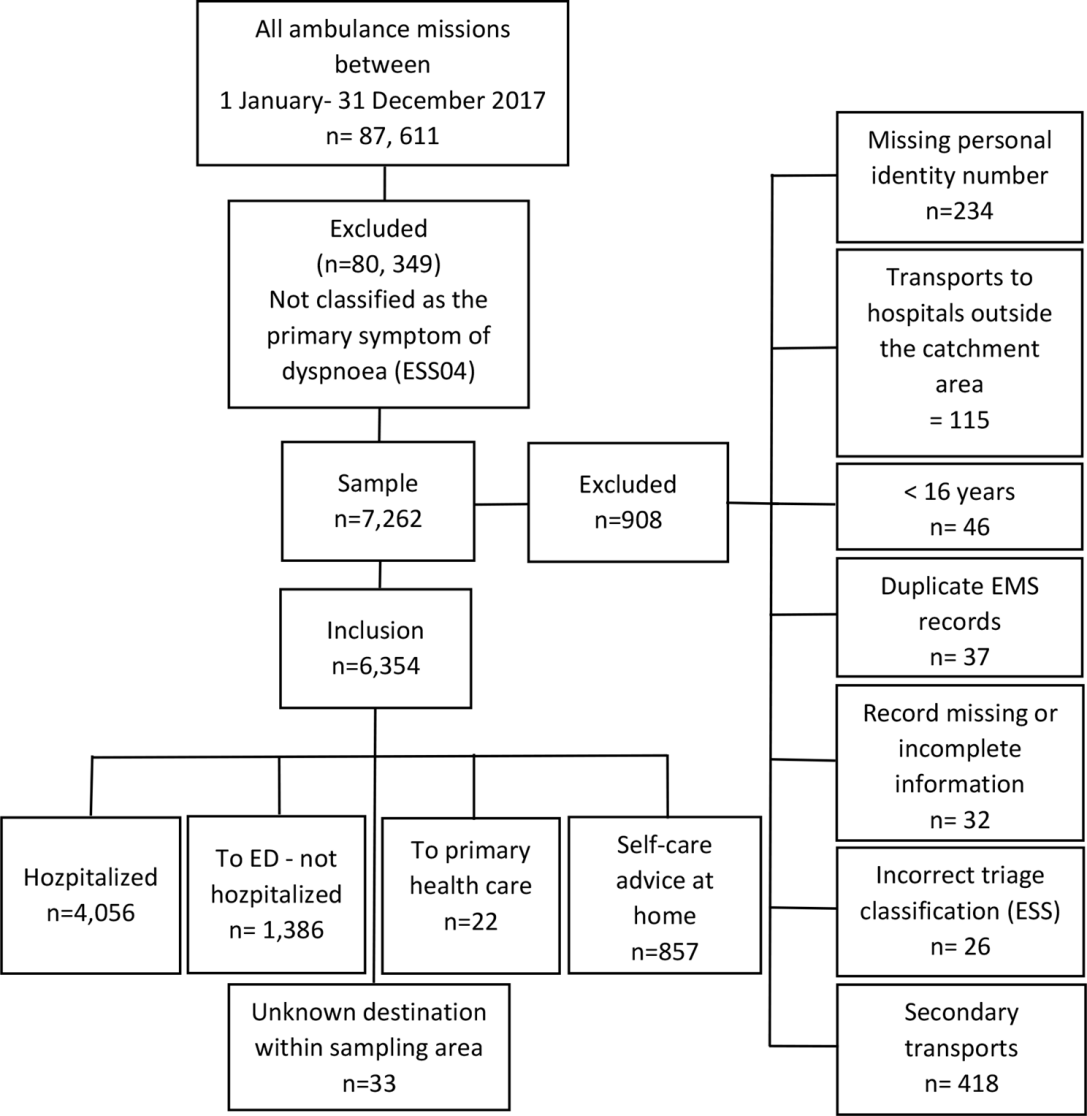
Flow chart of the studied patients, assessed with dyspnoea as the primary symptom

The study included two EMS organisations in the south-western part of Sweden with a population of 962,000 inhabitants and a catchment area of approximately 7,400 km^2^, including both urban, suburban, and rural areas. In all, the EMS organisations had 123,614 missions with a priority level of 1 to 3. Among them, 87,611 were considered a primary mission involving an initial patient assessment (Fig. 1; previously published in Kauppi et al.(2020) [2]). In Sweden, all ambulances are staffed with at least one registered nurse (RN) [9]. In addition, a major part of the RNs has fulfilled a postgraduate one-year master’s course with specialization in pre-hospital emergency care.

### Study population

Patients were recruited from two EMS organisations in Göteborg and Södra Älvsborg from 1 January to 31 December 2017. Inclusion criteria encompassed ambulance dispatches following contact with the Swedish emergency number (112) where the primary symptom assessed at the scene by EMS clinicians was dyspnoea, classified under Emergency Signs and Symptoms (ESS) number 04. ESS refers to a classification system used by EMS clinicians to assess and categorize patients based on presenting symptoms, such as dyspnoea. ESS number 04 specifically refers to cases where dyspnoea is the primary symptom assessed at the scene [19].

Exclusion criteria were the following: patient records with limited information, duplicate EMS records, patients under 16 years of age, no personal identity number, triaged with incorrect ESS- number, secondary transports and transported to a hospital outside the catchment area (Fig. 1).

### Predictors

The following predictors were evaluated: demographic variables age and sex, and the patient’s medical history of dyspnoea, ischemic heart disease, heart failure, hypertension, diabetes, atrial fibrillation, pulmonary disease, renal disease, systemic disease, cancer and psychiatric disorder were assessed. We also included symptoms of pain and syncope, along with VP recorded upon EMS clinicians’ admission. These VP encompass respiratory rate, oxygen saturation, systolic blood pressure, heart rate, body temperature, and the level of consciousness. Lastly, pathologic electrocardiogram (ECG), time from symptom onset until the call for an ambulance, time from the call for an ambulance until EMS clinicians’ arrival, and the patient’s total number of EMS visits were investigated.

### Traditional triage methods

For reference, we also evaluated RETTS-A [17] which is used in the majority of the EMS organisations in Sweden, as well as NEWS2 (score 1–20) [18], recommended for use in the EMS and hospital. RETTS-A utilizes both the VP and ESS independently to recommend a final priority level. The final triage level is determined by the highest level from either ESS or VP. The priority levels are color-coded (red, orange, yellow, green, and blue) to indicate the time from assessment until patients need to be seen by a physician. Red and orange signify the highest priority (life-threatening or potentially life-threatening), while the other colours indicate situations where delaying physician assessment has a lower medical risk. The blue colour is not used within the EMS [17]. The NEWS2 tool provides a cumulative numerical score through the assessment of VP and oxygen use, aiming to assess illness severity and identify potential SAE in high-risk patients [18]. Oxygen “level 2” score was not used in the evaluation according to recommendations [20].

### Outcome measures

The outcome of interest was a SAE, defined as any of the following: (1) death within 30 days after calling for an ambulance, (2) a final diagnosis defined as a time-sensitive diagnosis (e.g. myocardial infarction, pulmonary embolism and sepsis, which in order are the most frequent diagnoses [see Additional file 1], 3) admitted to an ICU, or 4) readmission within 72 h and admitted to hospital and received a final time- sensitive diagnosis.

### Statistical methods

Descriptive data are presented in numbers and percentages. In univariable analyses, the relative risk of a SAE was estimated using generalized estimating equations with Poisson regression and log-link, unadjusted and adjusted for age. Robust standard errors were used to account for multiple observations (i.e., EMS calls) from the same subject.

Multivariable prediction models were developed as follows: the original dataset was split into 80% training data and 20% test data. Model development and internal validation were performed on the training dataset using 10-fold cross-validation, and the best-performing model was subsequently evaluated on the test dataset. The prediction models considered included logistic regression with stepwise selection, the Least Absolute Shrinkage and Selection Operator (LASSO), two-stage LASSO, and gradient boosting trees. For comparison, RETTS-A and NEWS2 were also modeled using logistic regression. Hyperparameters of the ML models were optimized based on cross-validation deviance, and missing data were handled using stochastic imputation using random forests. Logistic regression and LASSO were selected as representative linear models due to their widespread use in clinical prediction tasks and their interpretability. LASSO, in particular, offers regularization to manage feature selection and mitigate overfitting. In contrast, gradient boosting trees were included as a nonlinear model capable of capturing complex relationships between predictors and outcomes, allowing for an assessment of whether such flexibility could improve performance in this setting.

Model performance was evaluated using McFadden’s R^2^ as measure of overall performance and goodness-of-fit. Discrimination was assessed using the Receiver Operating Characteristic (ROC) curve, Area Under the Curve (AUC) and Tjur’s discrimination index. The calibration of the predictions was evaluated using calibration plots, Calibration-In-the-Large (CIL), and Calibration Slope (CS). Variable importance was evaluated by Shapley values, using multivariable R^2^-decomposition for stepwise logistic regression according to the method by Lindeman, Merenda and Gold [21] and SHAP (Shapley Additive exPlanations) TreeExplainer [22, 23] for gradient boosting. The relative importance is presented as a percentage, summing up to 100%. Statistical analyses were performed with Python, version 3.11.5, using the Statsmodels 0.14.0, Scikit-learn 1.2.2, and shap version 0.44.0 libraries. Variable importance for logistic regression was evaluated using the domir package, version 1.1.1, in the R language and environment for statistical computing (R Core Team, Vienna, Austria), version 4.3.1. The study was conducted and reported in accordance with the Transparent Reporting of a multivariable prediction model for Individual Prognosis or Diagnosis **(**TRIPOD) guideline [24].

## Results

### Descriptive statistics

Baseline characteristics of the study cohort are presented in Table 1. A total of 6,354 cases of ambulance missions were included in the study, of which 1,118 (18%) were SAEs. Out of these, 500 (45%) suffered death within 30 days, 598 (50%) were classified with a time-sensitive diagnosis, and 311 (28%) patients were admitted to an ICU. Most patients (78%) were 65 years or older and 44% were male. Seventy eight percent (78%) had a history of dyspnoea, 47% history of pulmonary disease, 46% hypertension, and 30% had a history of heart failure.

**Table 1:** Descriptive statistics of the study cohort

Variable	Full analysis set (*n* = 6,354)	Serious adverse event	
		No (*n* = 5,236)	Yes (*n* = 1,118)
Serious adverse event	1,118 (17.6%)		
Death within 30 days	500 (44.7%)		
Time-sensitive diagnosis	598 (49.5%)		
Admitted to ICU	311 (27.8%)		
Age (years)			
< 65	1,406 (22.1%)	1,253 (23.9%)	153 (13.7%)
65–80	2,385 (37.5%)	1,937 (37.0%)	448 (40.1%)
> 80	2,563 (40.3%)	2,046 (39.1%)	517 (46.2%)
Male sex	2,816 (44.3%)	2,279 (43.5%)	537 (48.0%)
Medical history			
Dyspnoea	4,971 (78.2%)	4,142 (79.1%)	829 (74.2%)
Ischemic heart disease	1,642 (25.8%)	1,336 (25.5%)	306 (27.4%)
Heart failure	1,894 (29.8%)	1,515 (28.9%)	379 (33.9%)
Hypertension	2,948 (46.4%)	2,372 (45.3%)	576 (51.5%)
Diabetes	1,190 (18.7%)	934 (17.8%)	256 (22.9%)
Atrial fibrillation	1,838 (28.9%)	1,518 (29.0%)	320 (28.6%)
Pulmonary disease	2,966 (46.7%)	2,563 (48.9%)	403 (36.0%)
Renal disease	759 (11.9%)	590 (11.3%)	169 (15.1%)
System disease	393 (6.2%)	315 (6.0%)	78 (7.0%)
Cancer	1,226 (19.3%)	975 (18.6%)	251 (22.5%)
Psychiatric disorder	1,337 (21.0%)	1,151 (22.0%)	186 (16.6%)
Other symptoms			
Pain	1,402 (22.1%)	1,156 (22.1%)	246 (22.0%)
Syncope	77 (1.2%)	53 (1.0%)	24 (2.1%)
Vital parameters			
Respiratory rate < 8 or > 25 breath/min	3,029 (47.7%)	2,275 (43.4%)	754 (67.4%)
Oxygen saturation < 90%	2,197 (34.6%)	1,537 (29.4%)	660 (59.0%)
Systolic blood pressure ≤ 90 mmHg	195 (3.1%)	98 (1.9%)	97 (8.7%)
Heart rate < 40 or > 120 beats/min	716 (11.3%)	473 (9.0%)	243 (21.7%)
Body temperature < 35.0 or > 41.0 ºC	33 (0.5%)	13 (0.2%)	20 (1.8%)
Degree of consciousness (RLS) > 2	208 (3.3%)	87 (1.7%)	121 (10.8%)
Pathological ECG	2,646 (41.6%)	2,003 (38.3%)	643 (57.5%)
Time from symptom onset to call (h)			
≤ 12	2,022 (31.8%)	1,629 (31.1%)	393 (35.2%)
12–24	295 (4.6%)	245 (4.7%)	50 (4.5%)
24–48	508 (8.0%)	414 (7.9%)	94 (8.4%)
48–72	614 (9.7%)	507 (9.7%)	107 (9.6%)
> 72	2,477 (39.0%)	2,081 (39.7%)	396 (35.4%)
Time from call to EMS arrival (min)			
0–6	113 (1.8%)	86 (1.6%)	27 (2.4%)
6–12	1,500 (23.6%)	1,186 (22.7%)	314 (28.1%)
12–24	2,663 (41.9%)	2,208 (42.2%)	455 (40.7%)
> 24	1,999 (31.5%)	1,692 (32.3%)	307 (27.5%)
RETTS-A			
Green	995 (15.7%)	968 (18.5%)	27 (2.4%)
Yellow	1,621 (25.5%)	1,485 (28.4%)	136 (12.2%)
Orange	1,941 (30.5%)	1,615 (30.8%)	326 (29.2%)
Red	1,797 (28.3%)	1,168 (22.3%)	629 (56.3%)
RETTS-A ESS Dyspnoea			
Green	998 (15.7%)	971 (18.5%)	27 (2.4%)
Yellow	1,613 (25.4%)	1,476 (28.2%)	137 (12.3%)
Orange	1,930 (30.4%)	1,602 (30.6%)	328 (29.3%)
Red	1,778 (28.0%)	1,158 (22.1%)	620 (55.5%)
RETTS-A Vital signs			
Green	1,699 (26.7%)	1,618 (30.9%)	81 (7.2%)
Yellow	888 (14.0%)	796 (15.2%)	92 (8.2%)
Orange	1,685 (26.5%)	1,393 (26.6%)	292 (26.1%)
Red	2,029 (31.9%)	1,383 (26.4%)	646 (57.8%)
NEWS2			
≤ 5	2,498 (39.3%)	2,326 (44.4%)	172 (15.4%)
6–10	2,135 (33.6%)	1,706 (32.6%)	429 (38.4%)
11–15	423 (6.7%)	240 (4.6%)	183 (16.4%)
> 15	21 (0.3%)	5 (0.1%)	16 (1.4%)

Data are presented as numbers and percentages

Abbreviations: EMS, emergency medical services; ECG, electrocardiogram; ICU, intensive care unit; NEWS2, national early warning score 2; RETTS-A, rapid emergency triage and treatment system for adults

**Table 2:** Performance of prediction models by cross-validation on training data, and final model evaluated on test data

Model	McFadden’s pseudo-*R*²	Tjur’s pseudo-*R*²	ROC AUC	CIL*	CS†
**Training data (*n*** = **5,083)**					
RETTS-A	0.11 (0.08–0.14)	0.10 (0.09–0.11)	0.70 (0.67–0.73)	-0.00 *p* = 1.00	1.00 *p* = 0.93
NEWS2	0.11 (0.08–0.14)	0.11 (0.09–0.13)	0.71 (0.68–0.74)	0.00 *p* = 0.99	1.00 *p* = 0.94
LASSO logistic regression	0.16 (0.14–0.18)	0.17 (0.15–0.19)	0.78 (0.76–0.80)	0.00 *p* = 0.95	0.95 *p* = 0.23
Stepwise logistic regression	0.18 (0.16–0.20)	0.19 (0.17–0.21)	0.79 (0.78–0.80)	0.00 *p* = 0.92	0.94 *p* = 0.15
Gradient boosting trees	0.20 (0.17–0.23)	0.20 (0.17–0.23)	0.80 (0.78–0.82)	0.02 *p* = 0.69	1.03 *p* = 0.40
**Test data (** ***n*** = **1**,**271)**					
Gradient boosting trees	0.22 (0.18–0.27)	0.22 (0.18–0.25)	0.81 (0.78–0.84)	0.03 *p* = 0.76	1.09 *p* = 0.27

Estimate with 95% confidence interval is presented

*The p-value is assessing the hypothesis that the calibration-in-the-large (calibration intercept) = 0, i.e., no systematic over- or underestimation of the overall risk predictions

†The p-value is assessing the hypothesis that the calibration slope = 1, i.e., no systematic underfitting (predictions too close to the mean) or overfitting (predictions of high/low risk observations too extreme)

Abbreviations: AUC, area under the curve; CIL, calibration-in-the-large, CS, calibration slope;

LASSO, least angle shrinkage and selection operator; NEWS2, national early warning score 2;

RETTS-A, rapid emergency triage and treatment system for adults; ROC, receiver operating characteristic

### Risk factors for serious adverse events

**Fig. 2:**
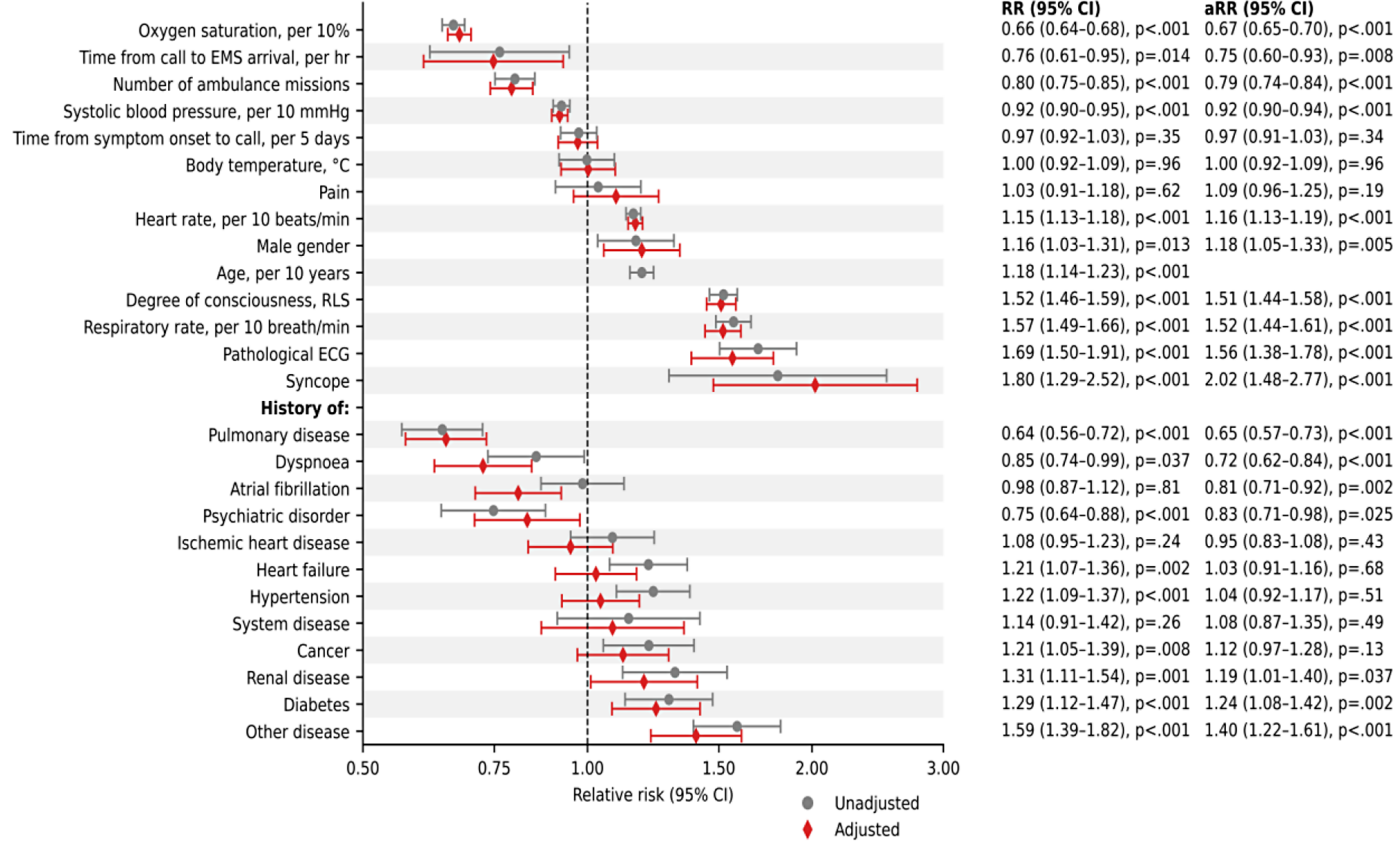
Crude and age-adjusted relative risk of serious adverse event in univariable analyses. aRR, adjusted relative risk; CI, confidence interval; RR, relative risk

**Fig. 3:**
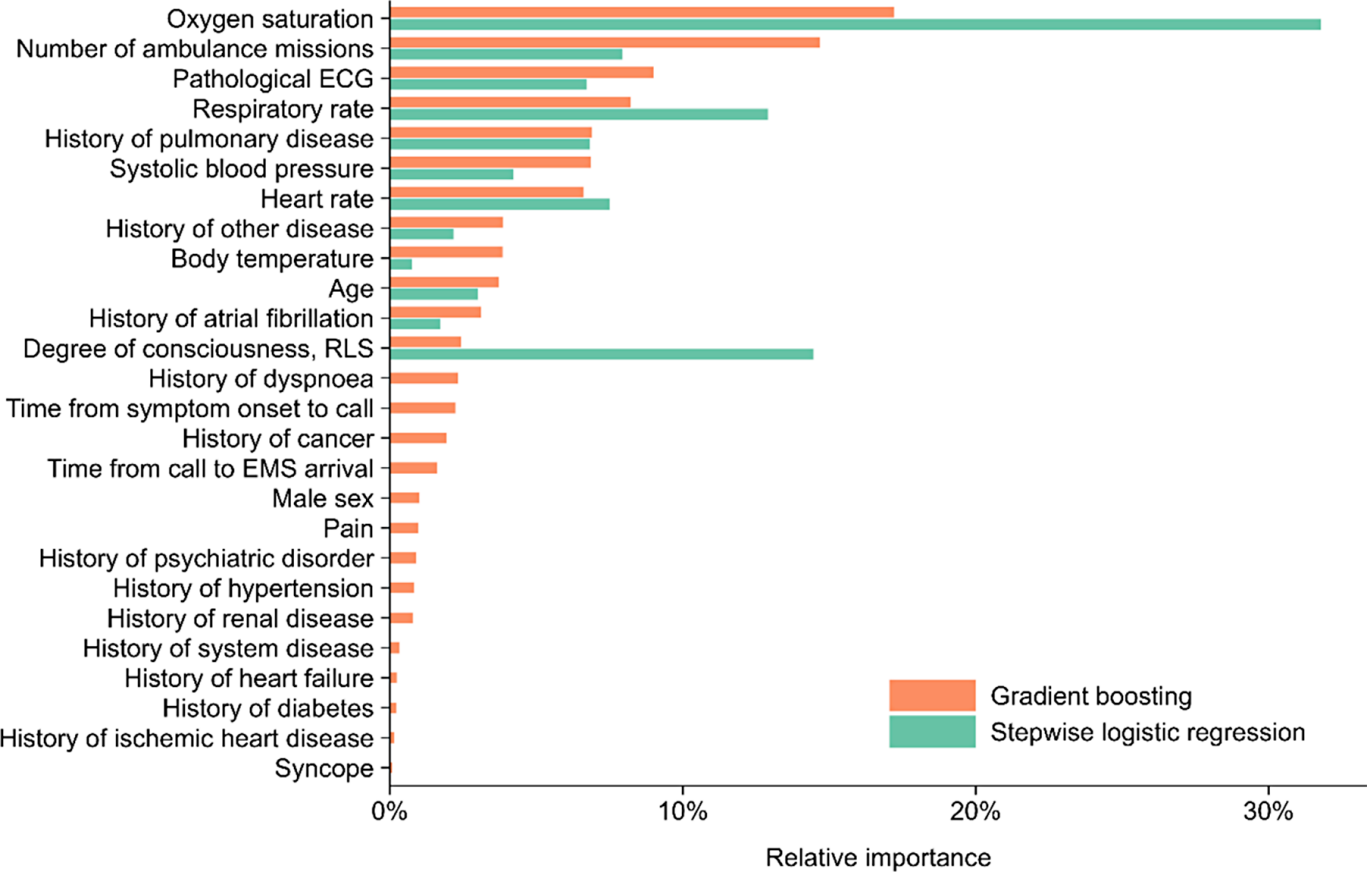
Variable importance using Shapley values according to gradient boosting and logistic regression. Bars are normalized to sum to 100%

A negative association was found between the risk of a SAE and low oxygen saturation, time from call to EMS clinicians’ arrival, number of ambulance visits, and a low systolic blood pressure (Fig. 2). A positive association was shown for abnormal heart rate, male sex, age, abnormal level of consciousness, abnormal respiratory rate, pathological ECG, and syncope. Regarding previous history, the risk of a SAE was lower among patients with a history of dyspnoea or pulmonary disease and higher among patients with a history of renal disease, diabetes, and other disease. In multivariable analyses, the seven most important variables in terms of gradient boosting feature importance in order were: low oxygen saturation, number of ambulance visits, pathological ECG, abnormal respiratory rate, history of pulmonary disease, low systolic blood pressure, and abnormal heart rate, together explaining 69% of the prognostic model (Fig. 3). In logistic regression analyses, the seven most important variable in order were: low oxygen saturation, abnormal degree of consciousness, abnormal respiratory rate, number of ambulance visits, abnormal heart rate, history of pulmonary disease, and pathological ECG.

### Model development and cross-validation performance

**Fig. 4:**
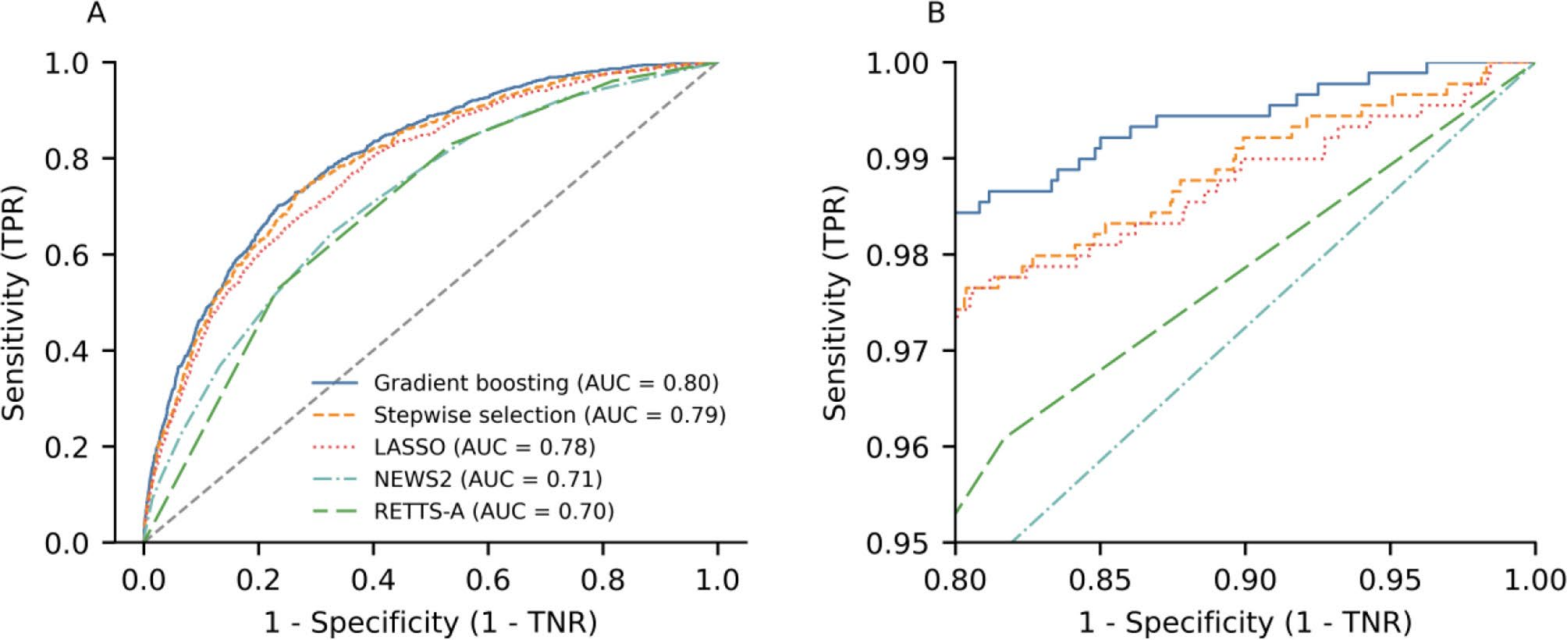
ROC curves for prediction of serious adverse events using ML and traditional triage methods. **A**: Full curve. **B**: Zoomed in at sensitivity ≥ 95% and 1 − specificity ≥ 80%. ROC, receiver operating characteristic; TNR, true negative rate; TPR, true positive rate

The performances of the proposed prediction models, internally validated through cross-validation, are presented in Table 2. All methods demonstrated strong calibration, with calibration intercepts close to 0 and slopes near 1, indicating a good match between predicted and observed outcomes. The prediction models consistently showed better performance than RETTS-A and NEWS2 across all performance metrics. Goodness-of-fit, measured by McFadden’s R², improved from 0.11 (95% CI 0.08–0.14) with RETTS-A/NEWS2 to 0.16 (0.14–0.18) with LASSO, 0.18 (0.16–0.20) with stepwise logistic regression, and 0.20 (0.17–0.23) with the gradient boosting model. Discrimination in terms of ROC AUC increased from 0.71 (0.68–0.74) to 0.78 (0.76–0.80) with LASSO, 0.79 (0.78–0.80) with stepwise logistic regression, and 0.80 (0.78–0.82) with gradient boosting. Similarly, Tjur’s discrimination index, which quantifies the mean difference in predicted risk between patients with and without SAE, increased from 0.10 (0.09–0.11) with RETTS-A/NEWS2 to 0.17 (0.15–0.19) with LASSO, 0.19 (0.17–0.21) with stepwise logistic regression, and 0.20 (0.17–0.23) with gradient boosting. The gradient boosting algorithm overall had the best performance and consistently showed higher sensitivity to detect SAE than the other methods, particularly in the high-sensitivity–low-specificity area of the ROC curve (Fig. 4).

**Table 3:** Risk stratification for prediction of severe adverse events using gradient boosting trees

Estimated risk	*n* obs.	*n* event (%)	True positive rate*	True negative rate*
**Training data** (***n*** = **5,083)**				
0.00–0.01	2	0 (0.0%)	1.00	0.00
0.01–0.02	89	0 (0.0%)	1.00	0.00
0.02–0.03	266	4 (1.5%)	1.00	0.02
0.03–0.04	316	6 (1.9%)	1.00	0.08
0.05–0.09	1452	86 (5.9%)	0.99	0.16
0.10–0.14	872	98 (11.2%)	0.89	0.48
0.15–0.19	538	88 (16.4%)	0.78	0.67
≥0.20	1548	612 (39.5%)	0.68	0.78
**Test data** (***n*** = **1**,**271)**				
0.00–0.01	0	0 (0.0%)	1.00	0.00
0.01–0.02	21	1 (4.8%)	1.00	0.00
0.02–0.03	76	2 (2.6%)	1.00	0.02
0.03–0.04	71	0 (0.0%)	0.99	0.09
0.05–0.09	372	17 (4.6%)	0.99	0.16
0.10–0.14	222	26 (11.7%)	0.91	0.50
0.15–0.19	141	25 (17.7%)	0.79	0.68
≥ 0.20	368	153 (41.6%)	0.68	0.79

*True positive rate and true negative rate evaluated with a cut-off at the lower endpoint of the estimated risk

### Model validation and risk stratification

**Fig. 5:**
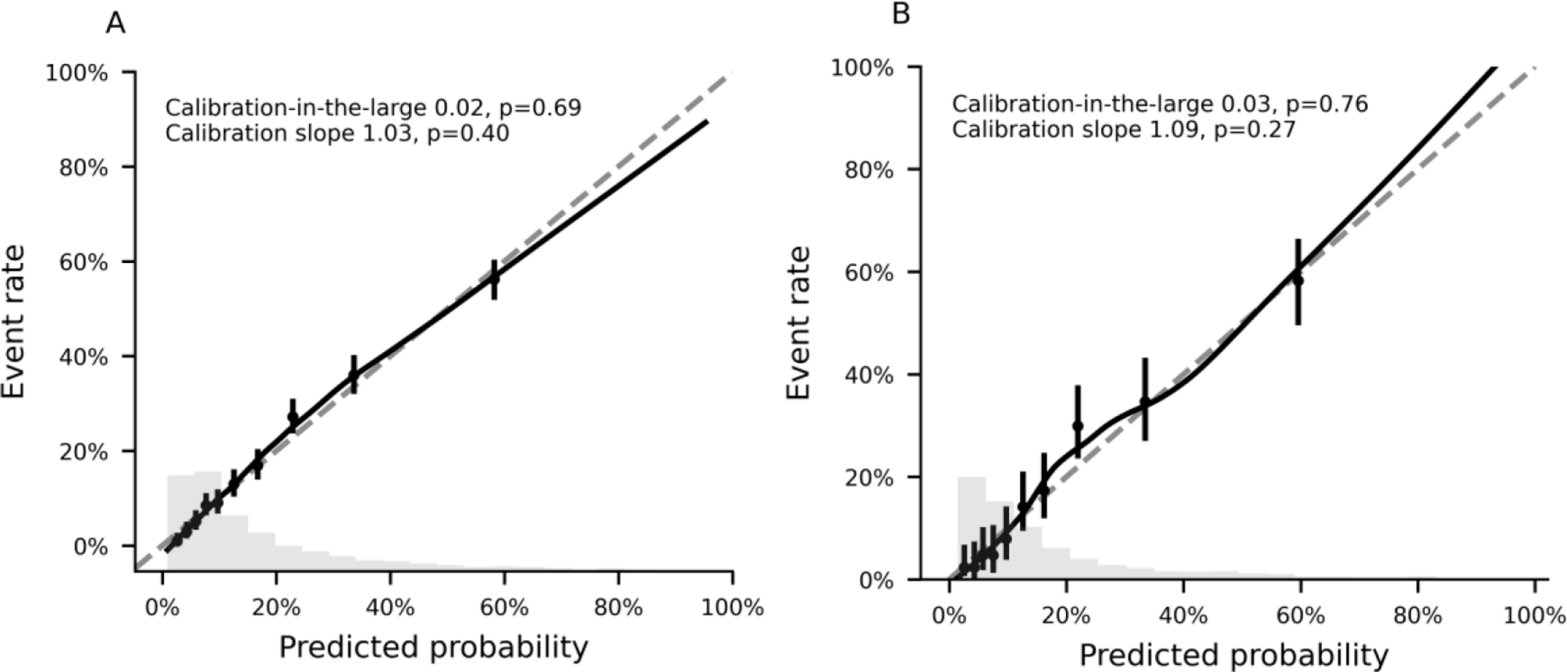
Calibration of gradient boosting for prediction of serious adverse events. **A**: Cross-validation performance on training data. **B**: Test data performance

The gradient boosting tree method was evaluated on the test data, demonstrating results consistent with its cross-validation performance on the training data. The model maintained a high goodness-of-fit, with McFadden’s R² (95% CI) at 0.22 (0.18–0.27), and good discrimination, achieving a ROC AUC of 0.82 (0.78–0.84) and a Tjur discrimination index of 0.22 (0.18–0.25). It also showed excellent calibration of the predictions to the observed event rates across the full range of predicted risks (Table 3; Fig. 5). At a 3% risk threshold, the model achieved a true positive rate (sensitivity) of 99% with a true negative rate (specificity) of 9%. Similarly, at a 10% risk threshold, the model achieved a true positive rate of 91% with true negative rate of 50%.

## Discussion

This study aimed to create an algorithm using clinical variables to predict a SAE among pre-hospital dyspnoeic patients. To our knowledge, this is the first study developing a decision support tool based on ML algorithms among patients assessed by EMS clinicians with the primary symptom of dyspnoea. The gradient boosting tree was the best model for predicting SAE and outperformed RETTS-A and NEWS2, which is a major and important finding. This in line with previous results reporting on superior performance when comparing different ML algorithms with NEWS2 on adverse outcomes such as hospital admission, admission to an ICU or mortality in general patient populations in both the EMS and the hospital [25–27]. A review involving unselected pre-hospital patients [28] revealed that NEWS2 demonstrates good sensitivity and specificity in predicting early death in pre-hospital settings and supports the utilization of NEWS2 as an initial assessment and triage tool for anticipating early death among pre-hospital patients overall. However, to enhance predictive accuracy, continuous patient monitoring with NEWS2 is recommended instead of solely relying on a single assessment.

Nevertheless, compared to ED and in-hospital care, the time where the patient is under care in the ambulance is short and most cases a continuous measurement over prolonged periods of time is not feasible, thus suggesting inclusion of readily available variables in the EMS besides VP to enhance the predictive ability. This implies that a model with additional variables other than present vital signs adds performance to the model. Gradient boosting trees and other ML approaches seem to perform well in the emergency setting [29–33]. Digital decision tools with the support of ML algorithms are considered feasible to use to aid the EMS clinician and thus have the potential to improve the pre-hospital assessment. An ML algorithm accounts for the specific patient presentation and, by that, could potentially capture more nuanced patterns and avoid loss of information, compared to instruments developed in-hospital with rough thresholds and sometimes arbitrarily chosen cut-offs. Although the gradient boosting tree model demonstrated good discriminatory performance, identifying a risk threshold that effectively balances high sensitivity and specificity is difficult, and accurately separating SAEs from non-SAEs remains challenging. This limitation may stem from the complexity of medical conditions, particularly those involving dyspnoea, which can be influenced by numerous factors [34]. In addition to potential diagnoses [2], factors such as genetics, lifestyle, and socioeconomic status which cannot be directly measured or observed through medical records may impact the prediction model. This suggests that these variables may interact with other factors in complex ways, contributing significantly to the model’s ability to predict outcomes. The importance of these predictors in the model highlights the intricate relationships between different health indicators, even if their direct effects are not significant on their own. In this study, SAE was defined as a binary variable based on four clinically significant indicators: death within 30 days of calling for an ambulance, a final diagnosis classified as time-sensitive, admission to an ICU, or readmission within 72 h followed by hospital admission with a final time-sensitive diagnosis. These indicators were selected for their strong association with acute deterioration and practical relevance to triage decision-making in EMS settings. Although patients with multiple simultaneous indicators may represent more severe conditions, a binary outcome (0/1) was chosen to align with standard triage practices, which focus on the presence or absence of critical events rather than their gradation. Future research could explore two alternative approaches to refine the outcome definition. First, treating SAE as an ordinal variable could capture varying degrees of severity by accounting for the number or type of indicators present, providing more detailed risk stratification for patients at heightened risk. Second, framing the problem as a multivariate prediction task with four separate binary outcomes would allow for a more granular understanding of how different predictors relate to specific adverse events. Both approaches offer the potential to enhance predictive accuracy and practical applicability in EMS settings and should be further investigated to determine their feasibility and impact on clinical decision-making.

Gradient boosting tree used in this analysis and multiple logistic regression used in our prior study can yield different results. Gradient boosting is an ML method capable of capturing complex, non-linear associations and interactions by combining an ensemble of weak predictors (e.g., decision trees) to create a strong predictor [35, 36]. However, the evaluated risk factors have been established previously [10]. The most important predictors appear to be reflected in the different VP, ECG findings, and medical history. Among them abnormal oxygen saturation [37–39] and low blood pressure [40–42] are well-known predictors of adverse outcomes. Our result also revealed that low blood pressure and low oxygen saturation play an important role in the overall performance of the model, influencing other predictors that contribute to the outcome. A history of pulmonary disease also emerged as an important predictor and may significantly impact the interplay of various health variables influencing outcomes. For instance, patients with chronic obstructive pulmonary disease (COPD) had an increased risk of cardiovascular events [43], linked to their comorbidities such as cardiovascular disease and/or chronic kidney disease, as well as a history of frequent exacerbations. Our results also highlighted the importance of the multiple ambulance visits in the overall model. One possible explanation for this is that a patient’s general health may influence the likelihood of experiencing a SAE. It is possible that other variables or factors not evaluated in the current study may be contribute the risk of a SAE in patients with dyspnoea and improve predictions further. Other factors, such as the severity of dyspnoea, may also be important for SAE prediction [44].

The ML model developed in this study differs from traditional triage systems such as RETTS-A and NEWS2 in the indicators it incorporates. RETTS-A and NEWS2 rely on standardized thresholds for vital signs and a limited set of variables, whereas the ML model integrates a broader range of clinical features, including patient history and additional physiological data. This broader scope enables the ML model to capture more complex relationships between predictors and outcomes, potentially improving predictive accuracy and risk stratification. However, these differences limit direct comparability, as the ML model is designed to explore the potential of incorporating additional data sources beyond standard triage indicators.

This study highlights the potential for ML-based prognostic models to transform EMS practice by improving risk stratification and supporting data-driven triage decisions. Advances in AI technology can enhance EMS clinicians’ ability to identify high-risk patients, optimize resource allocation, and improve patient outcomes. Future efforts should focus on validating AI/ML models across diverse settings, integrating them into existing workflows, and evaluating their broader impact on clinical care and system efficiency. Furthermore, efforts should focus on evaluating the practical implementation of ML models in real-time EMS workflows to determine their impact on patient outcomes and operational efficiency. Ensuring real-time functionality, usability, and adaptability to the dynamic EMS environment will be essential for successful implementation.

## Limitations

The study has several limitations, including the following:

1. The data was from 2017 and hence is relatively old. Although some aspects of disease outbreaks and treatment methods may have changed since 2017, most likely patients’ experiences of symptoms such as dyspnoea have not changed significantly. Dyspnoea is a fundamental symptom directly linked to the patient’s physical condition rather than external factors.
2. The data was confined to the southwest region of Sweden, which may restrict the generalizability of its findings to other geographical areas. Particularly, people in the northern part of Sweden face greater challenges in accessing EMS and longer transportation distances, potentially leading to variations in results. Thus, a validation of the ML models in other EMS organisations is recommended.
3. Due to the retrospective nature of data collection from medical records, there is a risk of incomplete documentation, which may have impacted the accuracy and reliability of the findings.
4. Dyspnoea as a symptom can manifest in various medical conditions besides those directly related to emergency situations, potentially leading to misclassification of patients.
5. There was missing information in medical records in 1,030 cases. This was mainly attributed to patients being left on the scene or discharged directly from the ED without a physician assessment. Despite efforts to reduce bias associated with medical record reviews, these missing data points could affect the overall validity of the study’s conclusions. However, this study followed the methodology outlined in a paper by Kaji et al. [45] aiming to reduce bias associated with medical record reviews.

## Conclusions

Using data from 6,354 ambulance missions caused by patients suffering from dyspnoea, a machine learning model using gradient boosting trees demonstrated good performance for predicting SAE, with substantial improvements over the more established decision support methods RETTS-A and NEWS2. Future research should focus on external validation in diverse EMS organisations, integration into real-time workflows, and evaluation of its impact on decision-making and patient outcomes.

## Electronic supplementary material

Below is the link to the electronic supplementary material.

Supplementary Material 1

## Data Availability

The dataset used during the current study are available from the corresponding author on reasonable request. The dataset is available at the University of Borås repository (registrator@hb.se). Persistent weblink to dataset: http://urn.kb.se/resolve?urn=urn:nbn:se:hb:diva-30670.

## Abbreviations

AI: Artificial Intelligence
AUC: Area Under the Curve
CIL: Calibration-In-the-Large
COPD: Chronic Obstructive Pulmonary Disease
CS: Calibration Slope
ECG: Electrocardiogram
ED: Emergency Department
EMS: Emergency Medical Services
ESS: Emergency Signs and Symptoms
ICU: Intensive Care Unit
LASSO: Least Angle Shrinkage and Selection Operator
ML: Machine Learning
NEWS2: National Early Warning Score 2
RETTS-A: Rapid Emergency Triage and Treatment System for Adults
RN: Registered Nurse
ROC: Receiver Operating Characteristic
SAE: Serious Adverse Event
TRIPOD: Transparent Reporting of a multivariable prediction model for Individual Prognosis or Diagnosis
VP: Vital Parameter
