# Supplementary material for: Advancing a machine learning-based decision support tool for pre-hospital assessment of dyspnoea by emergency medical service clinicians: a retrospective observational study
Source: BMC Emerg Med. 2025 Jan 5;25:2. doi: 10.1186/s12873-024-01166-9 (PMC11702062; doi:10.1186/s12873-024-01166-9)
Supplement: Supplementary file 1 — Supplementary Material 1 [file 12873_2024_1166_MOESM1_ESM.docx]

| **Additional file 1.** Occurrence of time-sensitive conditions  according to final diagnosis^4^ | | | | | | |  |  |  |  |
| --- | --- | --- | --- | --- | --- | --- | --- | --- | --- | --- |
|  | All patients |  |  |  |  |  |  |  |  |  |
| N | 6354 |  |  |  |  |  |  |  |  |  |
| Missing - n | 1030 |  |  |  |  |  |  |  |  |  |
|  | 5324 |  |  |  |  |  |  |  |  |  |
| **Cardiac** | **219 (4.1)** |  |  |  |  |  |  |  |  |  |
| Myocardial infarction | 127 (2.4) |  |  |  |  |  |  |  |  |  |
| Cardiac arrest | 1 (<0.1) |  |  |  |  |  |  |  |  |  |
| Pulmonary oedema | 83 (1.6) |  |  |  |  |  |  |  |  |  |
| Unstable angina | 8 (0.2) |  |  |  |  |  |  |  |  |  |
| **Vascular** | **130 (2.4)** |  |  |  |  |  |  |  |  |  |
| Pulmonary embolism | 123 (2.3) |  |  |  |  |  |  |  |  |  |
| Vessel embolism | 4 (0.1) |  |  |  |  |  |  |  |  |  |
| Aortic dissection | 1 (<0.1) |  |  |  |  |  |  |  |  |  |
| Aortic rupture | 2 (<0.1) |  |  |  |  |  |  |  |  |  |
| **Infection and inflammation** | **137 (2.6)** |  |  |  |  |  |  |  |  |  |
| Sepsis | 111 (2.1) |  |  |  |  |  |  |  |  |  |
| Epiglottitis | 1 (<0.1) |  |  |  |  |  |  |  |  |  |
| SIRS^1^ | 17 (0.3) |  |  |  |  |  |  |  |  |  |
| Other infection^2^ | 8 (0.2) |  |  |  |  |  |  |  |  |  |
| **Respiratory** |  |  |  |  |  |  |  |  |  |  |
| Acute respiratory insufficiency | 78 (1.5) |  |  |  |  |  |  |  |  |  |
| **Neurological** | **14 (0.3)** |  |  |  |  |  |  |  |  |  |
| Status epilepsia | 1 (<0.1) |  |  |  |  |  |  |  |  |  |
| Stroke | 11 (0.2) |  |  |  |  |  |  |  |  |  |
| TIA | 2 (<0.1) |  |  |  |  |  |  |  |  |  |
| **Other^3^** | **20 (0.4)** |  |  |  |  |  |  |  |  |  |
| ^1^ SIRS, Systemic inflammatory response syndrome. | |  |  |  |  |  |  |  |  |  |
| ^2^Pneumonitis, pericarditis, perotonitis, hemorrhagic fever | | |  |  |  |  |  |  |  |  |
| ^3^Various reasons: acidosis, acute kidney failures,  acute intoxications. | | | |  |  |  |  |  |  |  |
| ^4^ All missions, including patients with multiple occasions (*n*=598). | | |  |  |  |  |  |  |  |  |
|  |  |  |  |  |  |  |  |  |  |  |
